# Preexisting Genetic Background Primes the Responses of Human Neurons to Amyloid β

**DOI:** 10.3390/ijms26199804

**Published:** 2025-10-08

**Authors:** Adedamola Saidi Soladogun, Li Zhang

**Affiliations:** Department of Biological Sciences, The University of Texas at Dallas, 800 W Campbell Rd, Mail Stop RL11, Richardson, TX 75080, USA

**Keywords:** amyloid beta, Aβ, amyloid beta (Aβ), APOE, inflammatory responses, cell migration

## Abstract

The deposition of amyloid beta (Aβ) in the human brain is a hallmark of Alzheimer’s disease (AD). Aβ has been shown to exert a wide range of effects on neurons in cell and animal models. Here, we take advantage of differentiated neurons from iPSC-derived neural stem cells of human donors to examine its effects on human neurons. Specifically, we employed two types of neurons from genetically distinct donors: one male carrying APO E2/E2 (M E2/E2) and one female carrying APO E3/E3 (F E3/E3). Genome-wide RNA-sequencing analysis identified 64 and 44 genes that were induced by Aβ in M E2/E2 and F E3/E3 neurons, respectively. GO and pathway analyses showed that Aβ-induced genes in F E3/E3 neurons do not constitute any statistically significant pathways whereas Aβ-induced genes in M E2/E2 neurons constitute a complex network of activated pathways. These pathways include those promoting inflammatory responses, such as IL1β, IL4, and TNF, and those promoting cell migration and movement, such as chemotaxis, migration of cells, and cell movement. These results strongly suggest that the effects of Aβ on neurons are highly dependent on their genetic background and that Aβ can promote strong responses in inflammation and cell migration in some, but not all, neurons.

## 1. Introduction

Alzheimer’s disease (AD) is the primary cause of dementia. In the US, an estimated 6.9 million people age 65 and older are living with the disease currently [1]. It is estimated that in 2021, 57 million people had dementia worldwide [2]. Every year, nearly 10 million new cases occur. AD was first described by Alois Alzheimer in 1898 [3,4]. AD is characterized by the formation of extracellular amyloid β plaques and neurofibrillary tangles (NFT) in the intracellular environment, neuronal death, and synaptic loss [5,6]. These molecular and cellular changes ultimately lead to memory loss, cognitive decline, behavioral changes, and, ultimately, death [7,8]. AD cases can be classified into early onset (EOAD) and late onset (LOAD) [9]. Individuals with EOAD develop symptoms before the age of 65 years, and EOAD cases represent approximately 5–10% of all AD cases [10,11,12]. A total of 10–15% of EOAD cases show highly penetrant mutations in the amyloid beta precursor protein (APP), presenilin 1 (PSEN1) and presenilin 2 (PSEN2) genes [13,14]. LOAD cases, with two-thirds of them being women, develop in patients older than 65 years [8,9,15]. The apolipoprotein E gene (*APOE*) alleles are the strongest genetic risk factor for LOAD [16,17]. APOE transports lipids in the brain and is responsible for their internalization [18]. There are three *APOE* alleles: E2 is considered protective (the rarest allele, 5–10%), E3 is considered neutral (the most common allele, ~80%), and E4 increases LOAD risk (10–15%) [19]. The AD risk is dose-dependent. Additionally, global and local genetic ancestries, other genetic risk loci and environmental factors influence the risk [20].

The deposition of amyloid beta (Aβ) is associated with both EOAD and LOAD, although the topography of amyloid deposition and cognitive profiles differ [21,22,23]. Aβ is generated from the enzymatic processing of APP (Amyloid Precursor Protein) via the amyloidogenic pathway [8]. APP is a type I integral transmembrane glycoprotein with a small intracellular C-terminal domain, an Aβ peptide region, and a large extracellular N-terminal domain [24]. In the amyloidogenic pathway, APP is cleaved at the β-site by a β-secretase (BACE1), leading to the generation of a soluble beta-amyloid precursor protein and the CTF-β containing 99 amino acids. Subsequently, CTF-β is cleaved by γ-secretase to release the Aβ [8]. The accumulation of Aβ leads to the formation of amyloid plaques. In EOAD, mutations in APP, PSEN1, and PSEN2 genes may cause the formation of toxic forms of Aβ. In LOAD, APOE4 is known to enhance Aβ pathology [25]. Multiple lines of evidence indicate that APOE4 likely causes earlier, faster, and higher levels of Aβ deposition in the human brain [26,27,28,29].

A plethora of experimental studies have shown that soluble forms of Aβ oligomers exert strong neurotoxic effects, likely leading to AD pathology (for a review, see [30]). Additionally, both endogenous and synthetic Aβ form soluble Aβ oligomers and are cytotoxic in animal models and cell cultures (for a review, see [31]). Aβ oligomers have been shown to cause neurotoxicity and neurodegeneration via multiple mechanisms [32]. For example, Aβ oligomers can exert neurotoxicity by forming ion channel pores that disrupt intracellular calcium homeostasis and cause mitochondrial dysfunction and oxidative stress [33,34]. Additionally, Aβ oligomers can directly block hippocampal long-term potentiation (LTP) [35]. Aβ oligomers can induce hyperexcitation in sensitive neurons and promote a vicious cycle of hyperactivation [36]. The myriad neurotoxicity of Aβ oligomers has motivated the development of antibodies against Aβ for the treatment of AD, including lecanemab [37,38] and donanemab [39]. However, these antibodies have limited success in effectively treating AD. This may not necessarily indicate that Aβ is not a crucial factor causing AD. Rather, this may indicate the complexity of many genetic factors and AD-associated cell types that are affected by Aβ and that can affect Aβ levels and clearance [40]. Thus, it may be informative to examine and compare how Aβ oligomers influence neurons of different genetic backgrounds.

In this report, we took advantage of newly differentiated neurons from iPSC-derived neural stem cells of human donors. Those donors clearly possess different genetic background, including *APOE* alleles and sex. Previous studies of Aβ effects on neurons generally did not consider the various genetic background and heterogeneity of diverse cell and animal models in drawing conclusions [31]. iPSC-derived neural stem cells exhibit the properties of fetal-stage cells and are indistinguishable from ESCs in the expression of age-related markers due to iPSC rejuvenation [41,42]. Thus, newly differentiated neurons from iPSC-derived neural stem cells should not exhibit the effects of aging and environment that the donors experienced on many markers such as the level of epigenetic methylated DNA, transcriptome, and functional phenotypes [43]. Rather, these neurons should reflect the effects of genetic differences in somatic cells of donors from which iPSC cells are derived. Using genome-wide RNA sequencing (RNA-Seq) analysis, we characterized the effects of Aβ on the transcriptome of neurons derived from two genetically distinct, scarcely available iPSC-derived neural stem cell lines. Our results show that genetic background exerts a profound effect on neuronal responses to Aβ. This may have a strong implication in how to interpret data relating to studies of Aβ and AD etiopathogenesis.

## 2. Results

### 2.1. Genome-Wide RNA-Seq Analysis Identified a Substantial Number of Genes That Were Up- or Down-Regulated in the Same Manner During Neuronal Differentiation of Two Genetically Distinct iPSC Cell Lines

**Figure 1:**
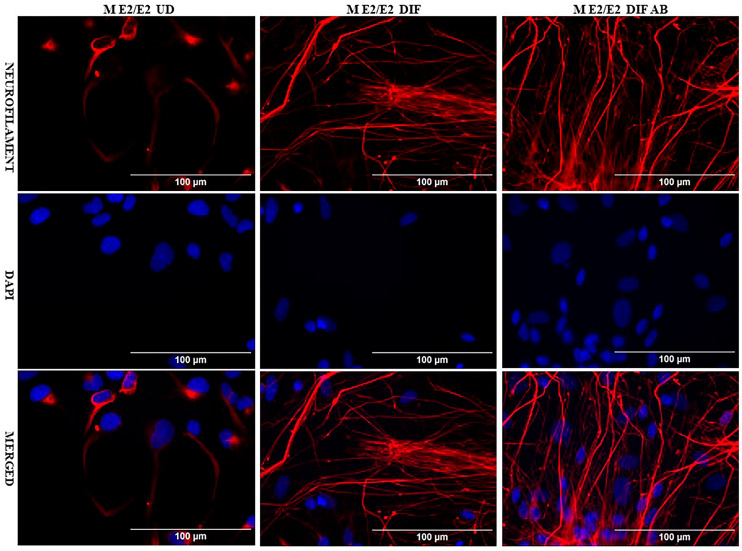
Immunocytochemistry images showing neurofilament protein NF-M in differentiated neurons derived from iPSC neural stem cells. Undifferentiated M E2/E2 iPSC cells (UD) or differentiated neurons (DIF) were stained with antibody to NF-M (red) or DAPI (blue). The images are shown here.

**Figure 2:**
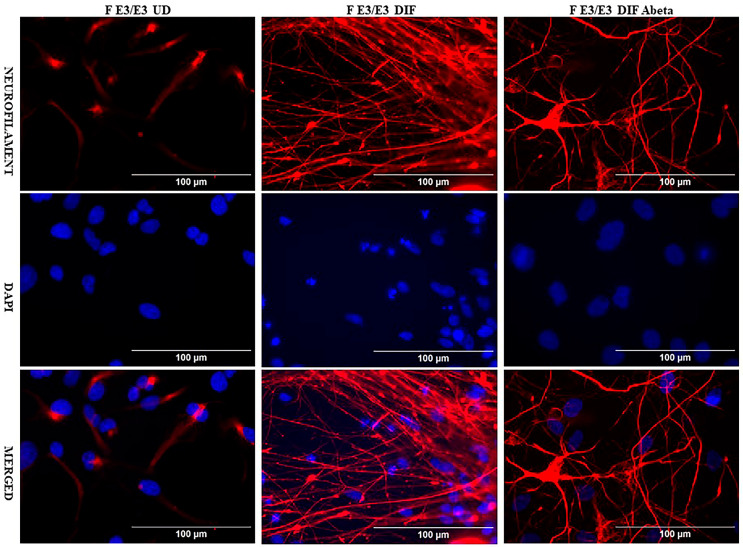
Immunocytochemistry images showing neurofilament protein NF-M in differentiated neurons derived from iPSC neural stem cells. Undifferentiated F E3/E3 iPSC cells (UD) or differentiated neurons (DIF) were stained with antibody to NF-M (red) or DAPI (blue). The images are shown here.

We acquired two scarcely available iPSC neural stem cell lines: one derived from a 74-year-old male carrying the APO E2/E2 allele (M E2/E2) and another derived from a 64-year-old female carrying the APO E3/E3 allele (F E3/E3). These cells were cultured and induced to undergo neuronal differentiation for 35 days. Then the cells were treated with oligomeric Aβ_42_ for 24 h. The cells were then examined with immunocytochemistry by staining with an antibody against the neuronal marker neurofilament protein (NF-M). Figure 1 and Figure 2 show the fluorescent images of M E2/E2 (Figure 1) and F E3/E3 cells (Figure 2). Aβ did not appear to have any disruptive effect on neurofilament formation.

Next, we prepare RNA from undifferentiated iPSC cells, differentiated neurons, and differentiated neurons treated with Aβ for RNA-Seq analysis. RNA-Seq data were then used to identify genes whose expression was changed in differentiated vs. undifferentiated iPSC cells and genes whose expression was changed by Aβ treatment. Briefly, genes with an adjusted false discovery rate of less than 0.05 in the given comparisons and with a log_2_FoldChange of greater than 0.8 or less than −0.8 were selected as differentially expressed genes. Table 1 summarizes the numbers of differentially expressed genes in relevant comparisons. As expected, there were a substantial number of genes whose expression was changed by neuronal differentiation in the same direction in both M E2/E2 and F E3/E3 cells. 357 genes were upregulated, while 526 genes were downregulated in both cell lines (see Common in Table 1).

Using the NIH DAVID GO analysis tool, we identified statistically significant GO groups for these up- and downregulated genes. Supplementary Tables S1 and S2 list the identified GO groups for up- and downregulated genes. Not surprisingly, the commonly upregulated genes include those involved in neurogenesis, clathrin-coated vesicle membrane, and synaptic vesicle membrane, as well as those involved in mitochondrial membrane (Supplementary Table S1). In contrast, many genes involved in cell cycle, mitosis, cell division, chromosome segregation, and DNA repair were commonly downregulated (Supplementary Table S2). This is in complete agreement with the fact that cell division and related processes are stopped upon neuronal differentiation. These results substantiated the validity of our RNA-Seq data.

### 2.2. Aβ-Induced Genes Are Predominantly Suppressed During Neuronal Differentiation

**Figure 3:**
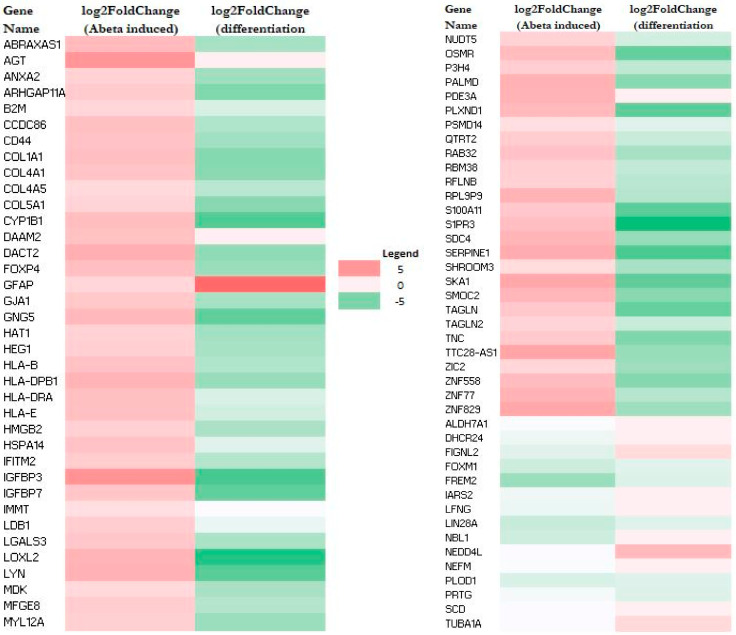
Heatmaps showing the patterns of transcript level changes induced by Aβ and neuronal differentiation in M E2/E2 neurons.

**Figure 4:**
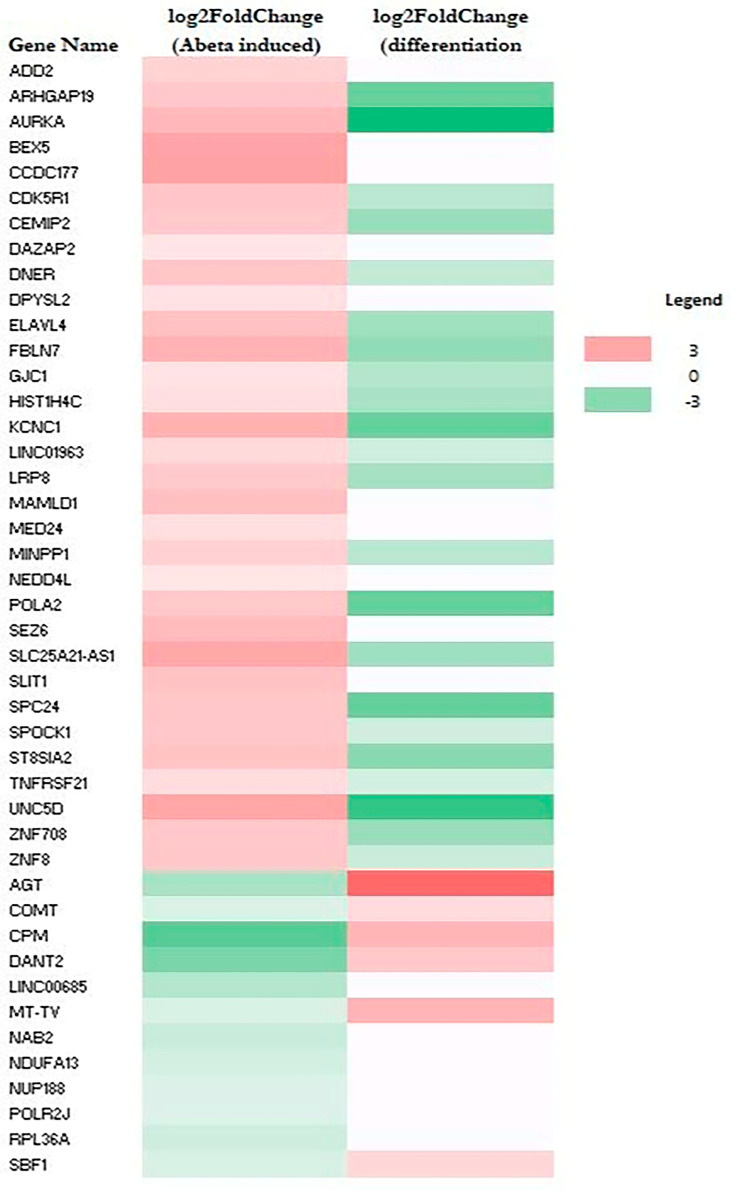
Heatmaps showing the patterns of transcript level changes induced by Aβ and neuronal differentiation in F E3/E3 neurons.

RNA-Seq data also allow the identification of genes whose expression was altered by Aβ. A total of 79 genes (upregulated: 64; downregulated: 15) and 44 genes (upregulated: 32; downregulated: 12) were altered in M E2/E2 and F E3/E3 neurons, respectively (Table 1). Interestingly, not one single gene was affected by Aβ in the same way in both types of neurons. Supplementary Tables S3 and S4 list all the genes whose expression was altered by Aβ in M E2/E2 and F E3/E3 neurons, respectively. The expression levels are also indicated by the heatmaps shown in Figure 3 and Figure 4.

Strikingly, all but one upregulated gene (a total of 63) induced by Aβ were downregulated or unchanged during neuronal differentiation (see Figure 3 and Supplementary Table S3). Likewise, all upregulated genes induced (a total of 32) by Aβ were downregulated or unchanged during neuronal differentiation (see Figure 4 and Supplementary Table S4). This suggests that Aβ has a counter effect on neuronal functions, as it induces many genes that are suppressed or not increased for supporting neuronal functions.

### 2.3. Pathway Analysis Shows That Aβ Induces a Network of Genes Promoting Inflammatory Responses and Cell Migration Selectively in M E2/E2 Neurons

To gain insights into the molecular mechanisms by which Aβ may influence neuronal functions, we carried out GO and pathway analysis. Using the NIH DAVID tool, we identified GO groups of Aβ-induced genes in neurons. Note that no statistically significant GO groups were identified for Aβ-suppressed genes. Supplementary Tables S5 and S6 list those statistically significant GO groups for induced genes in M E2/E2 and F E3/E3 neurons, respectively.

Many GO groups were identified for Aβ-induced genes in M E2/E2 neurons (Supplementary Table S5). These groups include the AGE-RAGE signaling pathway, ER to Golgi transport vesicle membrane, cell adhesion molecules, antigen processing and presentation, MHC class I protein complex, and so on. In contrast, only five overlapping GO groups were identified for Aβ-induced genes in F E3/E3 neurons (Supplementary Table S6). These results suggest that Aβ exerts stronger adverse effects on the functions of M E2/E2 neurons, compared to F E3/E3 neurons.

**Figure 5:**
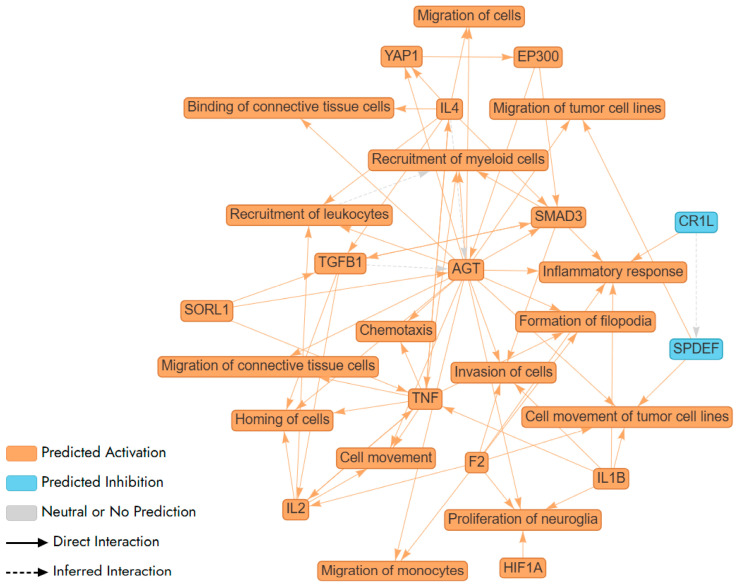
A network (illustrated via QIAGEN IPA Interpret) summarizing the statistically significant top predictions from a QIAGEN Ingenuity Pathway Analysis (IPA) Core Analysis of Aβ-regulated genes in newly differentiated neurons from a healthy male carrying APO E2/E2 alleles. AGT, F2, IL1B, and SMAD3 are shown to increase inflammatory responses, indicating a theme centered on the regulation of inflammation. This is further supported by the involvement of TNF, a key cytokine in inflammation, suggesting a complex network of interactions that modulate immune responses. AGT, IL2, and TNF enhance the recruitment of leukocytes and myeloid cells, highlighting a theme of immune cell mobilization. AGT, F2, HIF1A, and IL1B are linked to the proliferation of neuroglia, suggesting a theme of neural support and repair. This indicates the network’s involvement in maintaining and repairing neural tissues, which is essential for brain health and response to neural damage. The interactions involving SMAD3 and TGFB1 suggest a theme of signaling pathways that regulate various cellular processes, including inflammation, cell movement, and immune responses. These pathways are crucial for maintaining cellular homeostasis and responding to environmental changes.

Further, we used Ingenuity Pathway Analysis (IPA) tool to carry out pathway and network analysis. IPA did not identify any statistically significant pathway or network for Aβ-induced genes in F E3/E3 neurons. In contrast, IPA identified a complex network and multiple pathways involving Aβ-induced genes in M E2/E2 neurons (Figure 5). These genes included in every node/pathway in this network are listed in Supplementary Table S7. Clearly, this network involves two classes of pathways: those promoting inflammatory responses, such as IL1β/IL1B, IL4, and TNF/TNF-α; and those promoting cell migration and movement, such as chemotaxis, invasion of cells, and cell movement (Figure 5).

Table 2 lists Aβ-induced genes that are involved in TNF signaling (Benjamini–Hochberg *p* value: 3.1 × 10^−7^; *Z* score: 3.6), IL1β/IL1B (Benjamini–Hochberg *p* value: 2.0 × 10^−5^; *Z* score: 2.9), TGFβ1/TGFB1 (Benjamini–Hochberg *p* value: 1.8 × 10^−9^; *Z* score: 2.7), and IL4 (Benjamini–Hochberg *p* value: 0.004; *Z* score: 2.5). As shown in Table 2, 23 of 26 genes involved in TNF/TNF-α signaling were upregulated or downregulated, as expected if TNF signaling was activated; 16 of 17 genes involved in TNF signaling were upregulated, as expected if TNF signaling was activated; 25 of 29 genes involved in IL1β/IL1B signaling were upregulated or downregulated, as expected if IL1β/IL1B signaling is activated; 10 of 12 genes involved in IL4 signaling were upregulated, as expected if IL4 signaling was activated. Not surprisingly, these pathways are all involved in inflammatory responses and share common genes.

Table 3 lists Aβ-induced genes that are involved in migration of cells (Benjamini–Hochberg *p* value: 1.4 × 10^−8^; *Z* score: 3.3) and cell movement (Benjamini–Hochberg *p* value: 1.2 × 10^−8^; *Z* score: 3.2). Notably, 32 of 41 genes involved in migration of cells were upregulated or downregulated, as expected if migration of cells was activated; 35 of 43 genes involved in cell movement were upregulated or downregulated, as expected if cell movement was activated. Together, these results strongly suggest that signaling pathways involved in neuroinflammation and cell migration are preferentially activated in M E2/E2 neurons, not F E3/E3 neurons.

## 3. Discussion

Previous studies have well established that Aβ triggers neuroinflammation [44,45,46]. Aβ is a potent activator of microglia, leading to a change in microglial behavior and the release of a variety of proinflammatory and toxic molecules, including cytokines, such as IL-1β, and TNF, chemokines, reactive oxygen species, and nitric oxide [44,45,46]. Pro-inflammatory cytokines can then influence the expression and metabolism of the amyloid precursor protein (APP), leading to an increase in Aβ production. The neuroinflammation triggered by Aβ further promotes Aβ accumulation, which in turn causes more inflammation.

Here, our results show that Aβ activated the expression of many target genes of proinflammatory cytokines in M E2/E2 neurons (see Figure 5, Table 2, and Supplementary Table S7). This indicates that signaling by these proinflammatory cytokines are activated in neurons, not just microglia, by Aβ, although the activation appeared to occur only in certain type of neurons like M E2/E2 neurons, but not F E3/E3 neurons. This suggests that neurons of certain genetic background are highly sensitive to Aβ. Thus, in the human brains, neurons of certain genetic background like M E2/E2 neurons may suffer from inflammation caused by not only cytokines released by microglia, but also direct activation of cytokine signaling by Aβ. This may in part explains why accumulation of Aβ exerts varying toxicity in different humans.

Interestingly, our results also show that many gene involved in cell migration and movement were activated by Aβ selectively in M E2/E2 neurons (see Figure 5, Table 3 and Supplementary Table S7). Previous studies have shown that the amyloid precursor protein (APP), from which Aβ is derived, is crucial for the proper migration of neuronal precursor cells during brain development [47,48]. Additionally, previous studies have shown that APP could affect the migration and invasion of human breast cancer cells. Further, Aβ is known to promote the migration of blood cells including myeloid cells and lymphocytes [49]. Our results suggest that in certain neurons, Aβ, like APP, may also promote the migration and movement of neurons directly.

Our findings showing direct effects of soluble Aβ on select neurons of certain genetic background indicates that the effects of Aβ on neurons can be an early, initiating event for neuronal dysfunction, neurodegeneration, and AD pathogenesis only in patients of certain specific genetic background. This provides a specific mechanism leading to the highly heterogeneous nature of AD clinical presentation and pathogenic process [50,51,52,53].

## 4. Materials and Methods

### 4.1. iPSC Cell Culture and Differentiation

Two iPSC-derived neural stem cell lines were purchased from Axol Bioscience (Easter Bush Campus, Easter Bush, EH25 9RG, UK). They are ax0018: age 74 years old, male, APO E2/E2 (M E2/E2) and ax0019: 64 years old, female, APO E3/E3 (F E3/E3). Both were healthy individuals. The cells were cultured according to the manufacturer’s guidelines. Cells were maintained in a 5% CO_2_ atmosphere at 37 °C in tissue culture-grade 6-well plates coated with Surebond (Axol Bioscience). The culture medium, Neural Maintenance Media (Axol Bioscience) supplemented with EGF (20 ng/mL) and FGF2 (20 ng/mL), was refreshed every two days until the cells reached 70% confluence. Cells were then detached using Unlock (Axol Bioscience) and re-plated at a density of 70,000 cells/cm^2^.

For neuronal differentiation, iPSC-derived neural stem cells were seeded onto Surebond XF (Axol) pre-coated plates using Neural Plating Media (Axol Bioscience). After 24 h, the medium was changed to Neural Maintenance Media without EGF and FGF2. After an additional 24 h, the cells were switched to Neural Differentiation-XF Media, with media changes occurring every three days. On the sixth day, half of the medium was replaced with Neural Maintenance-XF Media (Axol Bioscience). Another 24 h later, half of the medium was again replaced with fresh Neural Maintenance-XF Media. The cells were cultured for a total of 35 days, with media changes every three days.

For preparing RNA from undifferentiated cells (UD), cells were collected prior to the initiation of neuronal differentiation process. For preparing RNA from differentiated neurons (DIF), cells were collected after the 35-day differentiation period.

For each cell line and condition, at least three sets of cells were collected and processed. An experimental workflow is shown below:




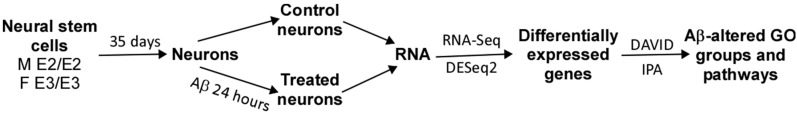




### 4.2. Oligomeric Aβ Preparation

Oligomeric Aβ was prepared following established procedures [54]. Aβ1–42 peptide (GenicBio, Shanghai, China) was dissolved in 1,1,1,3,3,3-hexafluoro-2-propanol (HFIP) to achieve a concentration of 1 mM, utilizing glass gas-tight Hamilton syringes with Teflon plungers. This solution was then divided into aliquots in micro centrifuge tubes and allowed to evaporate in a fume hood until dry. The dried peptide film was reconstituted in DMSO to a concentration of 5 mM and sonicated for 10 min in a bath sonicator. The peptide solution was then diluted with cold Neural Maintenance-XF Media to a final concentration of 100 mM and vortexed for 30 s. This solution was incubated at 4 °C for 24 h to facilitate oligomer formation. The oligomeric Aβ (0.5 mM) was then applied to neurons that had been differentiated for 35 days and incubated for 24 h before assays.

### 4.3. Immunocytochemistry

Immunocytochemistry was performed on neurons plated in 96-well plates following the established protocol [55]. The primary antibody used was an anti-NF M antibody (SYSY antibodies, catalog no. 171231). Cells were incubated with the primary antibodies overnight in a humidity chamber. Next, cells were rinsed three times for 5 min each with 2 mg/mL BSA in PBS. Cells were then incubated with the corresponding secondary antibodies, Alexa Fluor 488 (Thermo Fisher Scientific, Waltham, MA, USA, 1:100) or Alexa Fluor 594 (Thermo Fisher Scientific, 1:100), for 1 h at room temperature in a humidity chamber in the dark. For nuclear staining, cells were incubated with DAPI diluted in PBS (100 µL, 1:1000) for 5 min. After washing, 50 µL of Vectashield mounting medium was added to the cells, and cells were imaged with a Biotek Cytation 5 plate reader (DAPI 377/447; GFP 469/525; Texas Red 586/647).

### 4.4. RNA Sequencing (RNA-Seq) Analysis

iPSC neural stem cells were cultured in 6-well plates. Cells were collected at appropriate differentiation stages or after treatment with amyloid beta (Aβ). The collection involved scraping the cells, followed by centrifugation to remove PBS, and storing the samples at −80 °C for subsequent RNA sequencing analysis. For each cell line and condition, at least three sets of cells were collected and processed. Total RNA was extracted using the SPLIT Total RNA Kit (Lexogen, Vienna, Austria) following the manufacturer’s protocol. RNA integrity was assessed using an Agilent 2100 Bioanalyzer (Agilent Technologies, Santa Clara, CA, USA) with RNA integrity numbers (RIN) greater than 7 considered acceptable. RNA-seq libraries were prepared using the QuantSeq 3′ mRNA-Seq Library Prep Kit (Lexogen) according to the manufacturer’s instructions. Briefly, 40 ng of total RNA was used for library preparation, which included poly-A selection, cDNA synthesis, and library amplification with indexed primers. The libraries were sequenced on an Illumina NextSeq 2000 platform. The average input read lengths range from 87–93. Sequencing yielded an average of approximately 5 million reads per sample. The quality of raw sequencing data was assessed using FastQC (https://www.bioinformatics.babraham.ac.uk/projects/fastqc/, accessed on 10 August 2025). Adapter sequences and low-quality bases were trimmed using CUTADAPT.

Reads were aligned to the human reference genome (GRCh38) using STAR. The average mapping rate was approximately 85–90%. Differential gene expression analysis was conducted with DESeq2. Only genes with an adjusted *p*-value < 0.05 and log-fold change >0.8 or <−0.8 were selected as differentially expressed. RNA-Seq data are available at GEO database: accession number GSE288917.

### 4.5. Gene Ontology (GO) and Network Analysis of Transcriptomic Data

GO analysis was carried out using NIH DAVID (https://davidbioinformatics.nih.gov/summary.jsp, accessed on 10 August 2025). For further analysis of Aβ-altered genes, we employed QIAGEN’s Ingenuity Pathway Analysis (IPA). This is a powerful web-based software that helps the analysis of complex biological data in the context of biological pathways, networks, and functional annotations. Using this analysis tool, we were able to identify pathways and networks of Aβ-altered genes that are of statistical significance.

## 5. Conclusions

Aβ can directly exert inflammatory and cell movement effects on neurons with certain specific genetic background. One mechanism for Aβ to initiate neuronal dysfunction and AD pathogenesis is by directly eliciting inflammatory responses in neurons.

## Figures and Tables

**Table 1:**
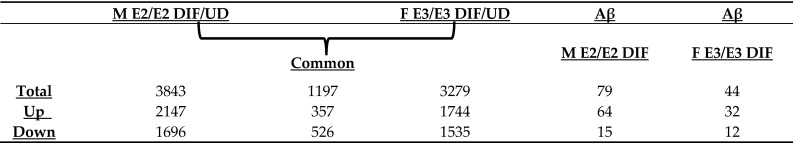
The number of genes whose expression is changed by Aβ or during neuronal differentiation.

**Table 2:** Lists of Aβ-induced genes that are targets of inflammatory pathways.

Name	log2FoldChange	Expected ^a^	Molecule Type	Location
TNF pathway				
AGT	5.05	Down	growth factor	Extracellular Space
B2M	1.4	Up	transmembrane receptor	Plasma Membrane
CD44	2.51	Up	other	Plasma Membrane
COL1A1	2.72	Up	other	Extracellular Space
COL4A1	2.4	Up	other	Extracellular Space
COL5A1	1.55	Up	other	Extracellular Space
CYP1B1	2.82	Up	enzyme	Cytoplasm
GFAP	1.34	Up	other	Cytoplasm
GJA1	2.14	- -	transporter	Plasma Membrane
HLA-B	2.57	Up	transmembrane receptor	Plasma Membrane
HLA-DRA	2.62	Up	transmembrane receptor	Plasma Membrane
HLA-E	2.63	Up	transmembrane receptor	Plasma Membrane
IFITM2	1.96	Up	other	Cytoplasm
IGFBP3	5.19	Up	other	Extracellular Space
LFNG	−1.35	Down	enzyme	Cytoplasm
LGALS3	2.22	Down	other	Extracellular Space
LOXL2	3.31	Up	enzyme	Nucleus
LYN	3.47	- -	kinase	Cytoplasm
NEFM	−0.92	Up	other	Plasma Membrane
OSMR	2.88	Up	transmembrane receptor	Plasma Membrane
RAB32	2.52	Up	enzyme	Cytoplasm
S1PR3	2.78	Up	G-protein coupled receptor	Plasma Membrane
SDC4	3.3	Up	other	Plasma Membrane
SERPINE1	3.82	Up	other	Extracellular Space
TAGLN	2.19	Down	other	Cytoplasm
TNC	2.13	Up	other	Extracellular Space
IL1β pathway				
ALDH7A1	−0.88	- -	enzyme	Cytoplasm
B2M	1.4	Up	transmembrane receptor	Plasma Membrane
CD44	2.51	Up	other	Plasma Membrane
COL1A1	2.72	Down	other	Extracellular Space
CYP1B1	2.82	Up	enzyme	Cytoplasm
DAAM2	2.6	Up	other	Cytoplasm
GJA1	2.14	Up	transporter	Plasma Membrane
HLA-B	2.57	Up	transmembrane receptor	Plasma Membrane
HLA-DRA	2.62	- -	transmembrane receptor	Plasma Membrane
HLA-E	2.63	Up	transmembrane receptor	Plasma Membrane
IFITM2	1.96	Up	other	Cytoplasm
IGFBP3	5.19	- -	other	Extracellular Space
LYN	3.47	Up	kinase	Cytoplasm
OSMR	2.88	Up	transmembrane receptor	Plasma Membrane
S1PR3	2.78	Up	G-protein coupled receptor	Plasma Membrane
SDC4	3.3	Up	other	Plasma Membrane
SERPINE1	3.82	Up	other	Extracellular Space
TGFβ1 pathway				
ANXA2	1.62	Up	other	Plasma Membrane
B2M	1.4	- -	transmembrane receptor	Plasma Membrane
CD44	2.51	Up	other	Plasma Membrane
COL1A1	2.72	Up	other	Extracellular Space
COL4A1	2.4	Up	other	Extracellular Space
COL5A1	1.55	Up	other	Extracellular Space
DHCR24	−1.37	Down	enzyme	Cytoplasm
FOXM1	−2.45	Up	transcription regulator	Nucleus
GFAP	1.34	Down	other	Cytoplasm
GJA1	2.14	Up	transporter	Plasma Membrane
HAT1	1.65	- -	enzyme	Nucleus
HLA-DRA	2.62	Up	transmembrane receptor	Plasma Membrane
IGFBP3	5.19	Up	other	Extracellular Space
IGFBP7	2.28	Up	transporter	Extracellular Space
LDB1	1.89	- -	transcription regulator	Nucleus
LGALS3	2.22	Down	other	Extracellular Space
LOXL2	3.31	Up	enzyme	Nucleus
PLOD1	−2.01	Up	enzyme	Cytoplasm
RFLNB	1.71	Up	other	Unknown
S100A11	2.18	Up	other	Cytoplasm
S1PR3	2.78	Up	G-protein coupled receptor	Plasma Membrane
SCD	−0.86	Up	enzyme	Cytoplasm
SDC4	3.3	- -	other	Plasma Membrane
SERPINE1	3.82	Up	other	Extracellular Space
SMOC2	3.23	- -	other	Extracellular Space
TAGLN	2.19	Up	other	Cytoplasm
TAGLN2	1.51	Up	other	Cytoplasm
TNC	2.13	Up	other	Extracellular Space
TUBA1A	−0.86	Up	other	Cytoplasm
IL4				
ANXA2	1.62	Up	other	Plasma Membrane
CCDC86	2.67	Up	other	Nucleus
CD44	2.51	Up	other	Plasma Membrane
COL1A1	2.72	Up	other	Extracellular Space
GJA1	2.14	Down	transporter	Plasma Membrane
HLA-DRA	2.62	Up	transmembrane receptor	Plasma Membrane
HLA-E	2.63	Up	transmembrane receptor	Plasma Membrane
LFNG	−1.35	Up	enzyme	Cytoplasm
LGALS3	2.22	Up	other	Extracellular Space
SERPINE1	3.82	Up	other	Extracellular Space
SKA1	4.05	Up	other	Nucleus
TNC	2.13	Up	other	Extracellular Space

^a^ The “Expected” column values indicate the direction of regulation (up or down) that the gene in the dataset should have if the relevant pathway is activated, based on the literature findings.

**Table 3:** Lists of Aβ-induced genes that are involved in cell migration.

	log2FoldChange	Expected ^a^	Molecule Type	Location
migration of cells				
AGT	5.05	Up	growth factor	Extracellular Space
ANXA2	1.62	Up	other	Plasma Membrane
ARHGAP11A	2.02	Up	other	Cytoplasm
CD44	2.51	Up	other	Plasma Membrane
COL1A1	2.72	Up	other	Extracellular Space
COL4A1	2.4	Up	other	Extracellular Space
COL5A1	1.55	Up	other	Extracellular Space
CYP1B1	2.82	Up	enzyme	Cytoplasm
DAAM2	2.6	Up	other	Cytoplasm
DACT2	3.62	Down	other	Cytoplasm
FIGNL2	−1.77	Down	other	Plasma Membrane
FOXM1	−2.45	Up	transcription regulator	Nucleus
FOXP4	2.72	Up	transcription regulator	Nucleus
GFAP	1.34	Up	other	Cytoplasm
GJA1	2.14	Up	transporter	Plasma Membrane
IGFBP3	5.19	Down	other	Extracellular Space
IGFBP7	2.28	Down	transporter	Extracellular Space
LDB1	1.89	- -	transcription regulator	Nucleus
LFNG	−1.35	Up	enzyme	Cytoplasm
LGALS3	2.22	Up	other	Extracellular Space
LIN28A	−2.61	Up	other	Cytoplasm
LOXL2	3.31	Up	enzyme	Nucleus
LYN	3.47	Up	kinase	Cytoplasm
MDK	1.24	Up	growth factor	Extracellular Space
MFGE8	1.96	Up	other	Extracellular Space
NBL1	−2.4	Down	other	Extracellular Space
NEDD4L	−0.86	Down	enzyme	Cytoplasm
OSMR	2.88	Up	transmembrane receptor	Plasma Membrane
PLOD1	−2.01	Up	enzyme	Cytoplasm
PLXND1	3.02	Up	transmembrane receptor	Plasma Membrane
S100A11	2.18	Up	other	Cytoplasm
S1PR3	2.78	Up	G-protein coupled receptor	Plasma Membrane
SCD	−0.86	Up	enzyme	Cytoplasm
SDC4	3.3	Up	other	Plasma Membrane
SERPINE1	3.82	Up	other	Extracellular Space
SKA1	4.05	Up	other	Nucleus
SMOC2	3.23	Up	other	Extracellular Space
TAGLN2	1.51	Down	other	Cytoplasm
TNC	2.13	Up	other	Extracellular Space
TUBA1A	−0.86	- -	other	Cytoplasm
ZIC2	1.36	Up	transcription regulator	Nucleus
Cell movement				
AGT	5.05	Up	growth factor	Extracellular Space
ANXA2	1.62	Up	other	Plasma Membrane
ARHGAP11A	2.02	Up	other	Cytoplasm
CD44	2.51	Up	other	Plasma Membrane
COL1A1	2.72	Up	other	Extracellular Space
COL4A1	2.4	Up	other	Extracellular Space
COL5A1	1.55	Up	other	Extracellular Space
CYP1B1	2.82	Up	enzyme	Cytoplasm
DAAM2	2.6	Up	other	Cytoplasm
DACT2	3.62	Down	other	Cytoplasm
FIGNL2	−1.77	Down	other	Plasma Membrane
FOXM1	−2.45	Up	transcription regulator	Nucleus
FOXP4	2.72	Up	transcription regulator	Nucleus
GFAP	1.34	Up	other	Cytoplasm
GJA1	2.14	Up	transporter	Plasma Membrane
HMGB2	1.73	Up	transcription regulator	Nucleus
IGFBP3	5.19	- -	other	Extracellular Space
IGFBP7	2.28	Down	transporter	Extracellular Space
IMMT	0.96	Down	other	Cytoplasm
LDB1	1.89	- -	transcription regulator	Nucleus
LFNG	−1.35	Up	enzyme	Cytoplasm
LGALS3	2.22	Up	other	Extracellular Space
LIN28A	−2.61	Up	other	Cytoplasm
LOXL2	3.31	Up	enzyme	Nucleus
LYN	3.47	Up	kinase	Cytoplasm
MDK	1.24	Up	growth factor	Extracellular Space
MFGE8	1.96	Up	other	Extracellular Space
NBL1	−2.4	Down	other	Extracellular Space
NEDD4L	−0.86	Down	enzyme	Cytoplasm
OSMR	2.88	Up	transmembrane receptor	Plasma Membrane
PLOD1	−2.01	Up	enzyme	Cytoplasm
PLXND1	3.02	Up	transmembrane receptor	Plasma Membrane
S100A11	2.18	Up	other	Cytoplasm
S1PR3	2.78	Up	G-protein coupled receptor	Plasma Membrane
SCD	−0.86	Up	enzyme	Cytoplasm
SDC4	3.3	Up	other	Plasma Membrane
SERPINE1	3.82	Up	other	Extracellular Space
SKA1	4.05	Up	other	Nucleus
SMOC2	3.23	Up	other	Extracellular Space
TAGLN2	1.51	Down	other	Cytoplasm
TNC	2.13	- -	other	Extracellular Space
TUBA1A	−0.86	- -	other	Cytoplasm
ZIC2	1.36	Up	transcription regulator	Nucleus

^a^ The “Expected” column values indicate the direction of regulation (up or down) that the gene in the dataset should have if the relevant process is activated, based on the literature findings.

## Data Availability

RNA-Deq data are available at GEO database: accession number GSE288917.

## Supplementary Materials

The following supporting information can be downloaded at: https://www.mdpi.com/article/10.3390/ijms26199804/s1.

## Abbreviations

The following abbreviations are used in this manuscript:

AD	Alzheimer’s Disease
NFT	Neurofibrillary Tangles
EOAD	Early Onset Alzheimer’s Disease
LOAD	Late Onset Alzheimer’s Disease
APP	Amyloid Precursor Protein
PSEN1	Presenilin 1
PSEN2	Presenilin 2
APOE	Apolipoprotein E gene/alleles
Aβ	Amyloid Beta
BACE1	Β-Secretase
iPSC	Induced pluripotent stem cells
RNA	RNA Sequencing
CTF-β	C-terminal domain fragment beta
ESC	Embryonic stem cells
